# Early Postoperative Nausea and Vomiting in Routine Clinical Practice: A Retrospective Observational Study of Associated Perioperative Factors and Prophylactic Practices

**DOI:** 10.4274/TJAR.2026.262558

**Published:** 2026-10-30

**Authors:** Onurcan Balık, Filiz Alkaya Solmaz, Mustafa Soner Özcan, Gökçe İşcan, Pakize Kırdemir

**Affiliations:** 1 Necmettin Erbakan University Faculty of Medicine, Department of Pain Medicine, Konya, Türkiye; 2 Süleyman Demirel University Faculty of Medicine, Department of Anaesthesiology and Reanimation, Isparta, Türkiye; 3 Süleyman Demirel University Faculty of Medicine, Department of Family Medicine, Isparta, Türkiye

**Keywords:** Postoperative nausea and vomiting, antiemetic prophylaxis, Apfel risk score, opioid use, total intravenous anaesthesia (TIVA), inhalation anaesthesia

## Abstract

**Objective:**

Postoperative nausea and vomiting (PONV) remains a common complication in the post-anaesthesia care unit (PACU) and negatively affects patient comfort and satisfaction. In routine clinical practice, identifying perioperative factors associated with early PONV may help optimize preventive strategies and guide anesthetic management. This study primarily aimed to evaluate PONV during the first postoperative hour and associated perioperative factors, and secondarily to describe PONV at later postoperative time points and antiemetic practices.

**Methods:**

This retrospective observational study evaluated adult patients who underwent elective surgery under general or regional anaesthesia from January to December 2021. After applying eligibility and complete-case criteria, 584 ASA I-III patients were included. Demographic characteristics, anesthetic techniques, surgical features, Apfel risk factors, antiemetic prophylaxis, rescue antiemetic use, and binary PONV data at postoperative hours 1, 6, 12, and 24 were assessed.

**Results:**

PONV occurred in 128 patients (22%) during the first postoperative hour and decreased at later postoperative time points. In univariate analyses, first-hour PONV was associated with general anaesthesia, inhalational anesthetic use, perioperative opioid use, female sex, non-smoking status, and perioperative medication-related variables (*P *< 0.05). The adjusted analysis suggested that early PONV in routine clinical practice reflects an overlapping, multifactorial pattern rather than the effect of a single dominant perioperative factor.

**Conclusion:**

First-hour PONV remained a clinically relevant problem in routine perioperative care despite the common use of prophylactic antiemetics. These findings highlight the importance of careful early postoperative monitoring and individualized risk-based preventive strategies in daily anaesthesia practice.

**Main Points**

• First-hour postoperative nausea and vomiting (PONV) remained a clinically relevant problem during routine perioperative care but decreased at later postoperative time points.

• Early PONV was associated with overlapping patient-related, anesthetic-related, and medication-related variables, supporting its multifactorial nature in daily clinical practice.

• These findings highlight the importance of careful early postoperative monitoring, standardized risk assessment, and individualized preventive strategies.

## Introduction

Postoperative nausea and vomiting (PONV) occurs in approximately 20-30% of all surgical patients and in 70-80% of those with specific risk factors.^1^ PONV may result in patient dissatisfaction, serious clinical complications, and economic losses. Patients undergoing surgery have described PONV as a worse experience than postoperative pain.^2^ PONV can cause clinically significant complications such as wound dehiscence, esophageal rupture, pneumothorax, and delayed discharge from the post-anaesthesia care unit (PACU).^3^

Guidelines state that the incidence of PONV can be reduced to 40% or lower through the use of antiemetics.^4^ Most guidelines advocate the use of risk-based PONV scoring systems, such as the Apfel score, which recommend antiemetic strategies based on stratified risk levels.^5, 6, 7^

Although randomized controlled trials have demonstrated the effectiveness of risk-based PONV scoring systems, their implementation in daily perioperative care remains variable. As a result, the incidence of early PONV and the prophylactic and therapeutic approaches currently used in routine practice are not always clearly characterized. A better description of current practice may help identify perioperative factors associated with early PONV and clarify where preventive strategies may be further optimized. In recent years, multimodal approaches to PONV prevention have gained popularity. Enhanced Recovery After Surgery protocols combine both pharmacological and non-pharmacological methods.^4, 8^ Strategies include avoiding emetogenic agents, such as inhalation anesthetics, and using propofol for anaesthesia induction and maintenance. Shortened preoperative fasting, preoperative carbohydrate loading, and adequate hydration have also been shown to be beneficial. The use of regional anaesthesia techniques—such as epidural analgesia or transversus abdominis plane blocks—can reduce postoperative opioid requirements and subsequently lower PONV incidence.^4, 8^ NSAIDs are also recommended as opioid-sparing agents. Combining two or more antiemetics has been shown to enhance efficacy. Dexamethasone has also been proven to reduce the prevalence of PONV.^8^

Effective PONV management should include risk assessment, application of preventive strategies, determination of appropriate antiemetic dose and timing, and establishment of a rescue treatment algorithm tailored to each patient.^4, 8^

Accordingly, the primary objective of this study was to evaluate PONV during the first hour and perioperative factors associated with its occurrence in routine clinical practice. The secondary objectives were to describe PONV at postoperative hours 6, 12, and 24 and to characterize prophylactic and rescue antiemetic practices used in the study population.

## Methods

This retrospective observational study, using routinely recorded clinical data, was conducted at the Department of Anesthesiology and Reanimation of Süleyman Demirel University Faculty of Medicine following approval by the Clinical Research Ethics Committee of Süleyman Demirel University (decision no: 34, date: 26.01.2022).

Between January and December 2021, a total of 4,500 patients underwent surgery under general or regional anaesthesia at our institution. Because this was a retrospective chart review, 1,000 records with accessible archived anaesthesia forms and hospital information system data were reviewed based on availability. Patients younger than 18 years of age, those undergoing emergency surgery, and those classified as American Society of Anesthesiologists (ASA) physical status IV or V were excluded. A complete-case analytical approach was applied, and records with missing data required for Apfel risk assessment, anaesthesia characteristics, perioperative medication use, or postoperative PONV follow-up were excluded. Of the 1,000 records reviewed, 416 were excluded because they did not meet the eligibility criteria or had incomplete perioperative or postoperative follow-up data. The final analysis included 584 patients who met the study criteria and had complete and verifiable datasets. Because the reasons for exclusion were not recorded as mutually exclusive categories during retrospective screening, the exact number of patients excluded for each criterion could not be reliably reconstructed. The patient selection process is summarized in Figure 1.

Patient medical files were retrieved from the hospital archive and the hospital information system using protocol numbers obtained from operating room records. The following data were collected on standardized case report forms: demographic characteristics, medical history, presence of PONV risk factors according to the Apfel scoring system, surgical characteristics, anaesthesia-related variables (type of anaesthesia, anesthetic agents used, duration of anaesthesia), perioperative medication use, postoperative analgesic requirements, and occurrence of nausea and vomiting at postoperative hours 1, 6, 12, and 24. When nausea or vomiting was observed, the antiemetic treatments administered were also recorded. Preoperative fasting duration and type of surgery were extracted from the medical records and evaluated as perioperative variables. Time to postoperative oral intake was not consistently available in the retrospective records; therefore, it could not be included in the analysis.

For the purposes of this study, PONV was defined as the presence of nausea, vomiting, or both, as documented in postoperative follow-up records. PONV was evaluated at postoperative hours 1, 6, 12, and 24. The primary outcome was PONV during the first postoperative hour. Secondary outcomes included PONV at postoperative hours 6, 12, and 24 and use of rescue antiemetic treatment. No validated severity grading scale for nausea or vomiting was available in the retrospective records; therefore, PONV was analyzed as a binary outcome at each postoperative time point.

Personal data were anonymized, and all patient information was stored digitally without disclosure of individual identities. Written informed consent, obtained at hospital admission, referred to anaesthesia and routine clinical care. Because this was a retrospective chart review study using anonymized data, study-specific informed consent was not obtained. The retrospective use of anonymized patient data was approved by the Clinical Research Ethics Committee of Süleyman Demirel University.

### Statistical Analysis

Statistical analyses were performed using the Statistical Package for Social Sciences (SPSS) version 26 (SPSS Inc., Chicago, IL, USA). Qualitative variables were expressed as numbers and percentages, while quantitative variables were presented as mean ± standard deviation. The Kolmogorov-Smirnov and Shapiro-Wilk tests were used to assess the normality of the distribution of continuous variables.

For comparisons between two independent groups, the Student’s t-test was used for normally distributed variables and the chi-square test was used for categorical variables. Time-dependent changes in postoperative assessments 1, 6, 12, and 24 hours were evaluated using repeated-measures analysis of variance.

Associations between PONV and perioperative variables were first evaluated using univariate analyses at each postoperative time point. To address potential confounding, a multivariable logistic regression analysis was performed for first-hour PONV, the primary outcome. Variables considered clinically relevant were selected for adjusted analysis and entered into the model, including sex (female), smoking status (non-smoking), perioperative opioid use, type of anaesthesia (inhalational or total intravenous), and antiemetic prophylaxis regimen. The antiemetic prophylaxis regimen was entered as a categorical variable, with no antiemetic prophylaxis as the reference category. Because antiemetic agents were administered in overlapping combinations in routine clinical practice, prophylaxis was modeled as mutually exclusive regimens rather than as separate drug variables. Rare or overlapping prophylaxis regimens were retained as a separate category because individual rare combinations had very small cell counts. The Apfel risk score was not included in the same model as its individual components to avoid collinearity. Adjusted odds ratios (ORs) with 95% confidence intervals (CIs) were reported. A *P* value of <0.05 was considered statistically significant.

## Results

During the study period, 4,500 patients underwent surgery under general or regional anaesthesia at our institution. As shown in 1">Figure 1, 1,000 records with archived and accessible anaesthesia forms and hospital information system data were reviewed. After applying eligibility and complete-case criteria, 416 records were excluded, leaving 584 patients with complete and verifiable datasets for the final analysis.

The demographic characteristics, ASA physical status classifications, and perioperative features of the study population are summarized in Table 1. The distribution of surgical procedures is presented in Table 2. According to the surgical risk classification, 286 patients were categorized as low-risk, 263 as medium-risk, and 35 as high-risk (Table 1). General anaesthesia was administered to 461 patients, while 123 underwent surgery under regional anaesthesia (Table 1). Inhalational anesthetic agents were used in the majority of patients, whereas total intravenous anaesthesia (TIVA) was applied in a small proportion of cases (Table 3).

The distribution of PONV risk factors according to the Apfel scoring system is shown in Table 3. Overall, 358 patients had an Apfel risk score of 2 or higher (Table 3). Preoperative fasting duration and characteristics of perioperative oxygen administration are presented in Table 3. Preoperative carbohydrate loading was performed in a limited number of patients.

The distribution of mutually exclusive antiemetic prophylaxis regimens is summarized in Table 4. Overall, 467 patients received at least one prophylactic antiemetic regimen, while 117 patients did not receive antiemetic prophylaxis.

The incidence of PONV and the use of rescue antiemetics at different postoperative time points are shown in Table 5. The incidence of PONV was highest during the first postoperative hour, affecting 128 patients (22.0%), and decreased at subsequent postoperative time points, with 47 patients at 6 hours, 14 patients at 12 hours, and 7 patients at 24 hours (Table 5).

Because first-hour PONV was the primary outcome, perioperative factors associated with this early recovery period were examined in detail. Univariate analyses showed that first-hour PONV was related to patient-related factors, anesthetic technique, and perioperative medication exposures rather than to a single variable (Table 6). This pattern was consistent with the highest PONV incidence observed during the first postoperative hour and decreasing thereafter.

In the multivariable logistic regression analysis, perioperative opioid use remained significantly associated with first-hour PONV after adjustment for clinically relevant variables and antiemetic prophylaxis regimens (adjusted OR: 0.58, 95% CI: 0.38-0.89, *P*=0.013). Female sex, non-smoking status, general anaesthesia, inhalational anaesthesia, TIVA, and antiemetic prophylaxis regimens were not independently associated with first-hour PONV in the adjusted model. The inverse association observed for perioperative opioid use should be interpreted cautiously because opioid administration was not standardized and may reflect clinical selection in this retrospective cohort (Table 7).

Clinical and perioperative characteristics of patients who experienced PONV during the first postoperative hour are summarized in Panel B of Table 6. These descriptive findings provide additional context for the early PONV subgroup, while adjusted associations are presented separately in Table 7.

## Discussion

PONV remains a common complication following anaesthesia and adversely affects patient comfort and satisfaction. In some patients, PONV may lead to clinically significant consequences, particularly in those who cannot tolerate increases in heart rate, blood pressure, intrathoracic pressure, or central venous pressure. Previous studies have reported PONV incidences of up to 80% in high-risk populations and approximately 20-30% in the general surgical population.^1, 4^ In the present study, the incidence of nausea and vomiting during the first postoperative hour was 22%, which is consistent with rates reported in the literature. This finding suggests that early PONV remains a clinically relevant problem under routine perioperative conditions despite the widespread use of preventive strategies.

The incidence of PONV decreased progressively after the first postoperative hour, with lower rates observed at the 6^th^, 12^th^, and 24^th^ postoperative hours. This temporal pattern may reflect the decreasing emetogenic effect of anesthetic agents after early recovery, the administration of rescue antiemetics to symptomatic patients, and the stabilization of postoperative pain and analgesic requirements over time. Therefore, the first postoperative hour appears to represent a clinically important period for detecting early PONV and evaluating immediate prophylactic effectiveness in the PACU setting.

An important component of PONV management is identifying patients who are more likely to experience this complication. Numerous patient-related, surgical, and anesthetic factors have been associated with an increased risk of PONV. Patient-related factors such as female sex, non-smoking status, a history of motion sickness, and perioperative opioid use have been consistently reported, whereas the influence of surgical type and extent remains variable across studies.^4, 5, 6, 9, 10^

In the present study, first-hour PONV was associated with several patient-related, anesthetic-related, and medication-related factors in univariate analyses. However, after adjustment for clinically relevant variables, including antiemetic prophylaxis regimens, female sex, non-smoking status, general anaesthesia, inhalational anaesthesia, TIVA were not independently associated with first-hour PONV. Although perioperative opioid use remained statistically associated with first-hour PONV in the adjusted model, the inverse association should be interpreted cautiously, because opioid administration was not standardized and may reflect clinical selection rather than a causal or protective effect.

Perioperative opioid administration is a well-recognized contributor to PONV, and opioid-sparing analgesic strategies form an important component of multimodal perioperative care.^11, 12^ In the present study, perioperative opioid use remained statistically associated with first-hour PONV in the adjusted model; however, the inverse direction of this association should not be interpreted as a protective effect. Because opioid type, dose, timing, pain intensity, and rescue analgesic requirements were not standardized in the retrospective records, this finding may reflect clinical selection or unmeasured confounding. Therefore, detailed prospective studies are needed to clarify the relationship between opioid exposure and early PONV.

Risk stratification tools, such as the Apfel scoring system, are widely used to estimate the likelihood of PONV and guide prophylactic strategies.^5^ In the present study, a substantial proportion of patients had Apfel risk scores of two or higher, indicating a moderate-to-high baseline risk. Although the overall incidence of early PONV was relatively modest, the observed associations between Apfel-related factors and PONV are in line with previous literature.^5, 6^

The type and extent of surgery have been variably associated with PONV in prior studies. While certain procedures, such as laparoscopic and gynecologic surgeries, have been described as higher risk, evidence remains inconsistent.^4, 6^ In the present study, no strong association was observed between surgical type or extent and early PONV, suggesting that anesthetic and patient-related factors may play a more prominent role in this setting.

Dexamethasone and 5-HT_3_ receptor antagonists are commonly used antiemetic agents with established efficacy in PONV prophylaxis.^4, 13, 14, 15^ Combination antiemetic therapy has been shown to provide greater protection than single-agent prophylaxis.^4, 8, 13, 14^ In the adjusted model, antiemetic prophylaxis regimens were evaluated as mutually exclusive categories; however, none of the prophylaxis regimens was independently associated with first-hour PONV. Because prophylaxis was not randomized and was likely influenced by baseline risk and clinician preference, these findings should be interpreted as descriptive associations rather than direct evidence of prophylactic efficacy.

The main contribution of this study is its description of early PONV and antiemetic practice patterns in an unselected real-world surgical cohort rather than in a protocol-driven trial population. By evaluating PONV at multiple postoperative time points and combining univariate findings with an adjusted analysis of first-hour PONV, the study provides insight into how established risk factors and routine prophylactic practices manifest in daily anaesthesia care. These findings may help identify areas where standardized risk assessment, documentation of antiemetic timing, and opioid-sparing strategies could be improved in prospective studies.

Overall, the findings of this study reflect real-life perioperative practice and highlight the multifactorial nature of PONV. Given the retrospective design and the presence of potential confounding factors, the results should be interpreted as associative rather than causal. Nevertheless, these observations may help inform preventive strategies and reinforce the importance of individualized PONV management.^4, 6^

### Study Limitations

This study has several limitations that should be considered when interpreting the findings. First, its retrospective design inherently limits control over data completeness and the standardization of variables. A complete-case analytical approach was applied, and patients with missing data in key perioperative variables or postoperative follow-up were excluded. As a result, selection bias cannot be fully excluded, and the analyzed cohort may not entirely represent the source population. In addition, the 1,000 reviewed records were selected based on record availability rather than random sampling, which should be considered when interpreting the external validity of the findings.

Second, although a multivariable logistic regression analysis, including clinically relevant variables and antiemetic prophylaxis regimens, was performed for first-hour PONV, the retrospective design and the routinely recorded nature of the data limited the ability to control for all potential confounders. Antiemetic prophylaxis was not randomized and may have been influenced by baseline PONV risk, clinician preference, and intraoperative clinical decisions. In addition, important exposure details, including antiemetic timing, opioid type, opioid dose and timing, pain intensity, rescue analgesic requirements, and standardized severity grading of nausea or vomiting, were not consistently available. Therefore, the adjusted results, particularly those related to opioid use and prophylaxis regimens, should be interpreted as exploratory and associative rather than causal or predictive.

Third, the study reflects real-world clinical practice at a single center, which may limit the generalizability of its results to other institutions with different patient populations or perioperative management protocols. Additionally, the small number of patients receiving TIVA limited the interpretability of findings related to anesthetic maintenance techniques; these results should not be interpreted as evidence against the established antiemetic advantages of propofol-based anaesthesia.

Despite these limitations, the study provides valuable insight into the incidence of early PONV and associated perioperative factors in routine anaesthesia practice.

## Conclusion

This retrospective observational study showed that first-hour PONV remained a clinically relevant problem in routine perioperative care despite the common use of prophylactic antiemetics. Early PONV was associated with overlapping patient-related, anesthetic-related, and medication-related factors, supporting its multifactorial nature in daily anaesthesia practice. After adjustment, many of the observed associations were attenuated, emphasizing the need for cautious interpretation of retrospective real-world data. These findings highlight the importance of careful early postoperative monitoring, standardized risk assessment, and individualized risk-based preventive strategies.

## Ethics

**Ethics Committee Approval:** This study was approved by the Süleyman Demirel University Clinical Research Ethics Committee (decision no: 34, date: 26.01.2022).

**Informed Consent:** Written informed consent, obtained at hospital admission, referred to anaesthesia and routine clinical care. Because this was a retrospective chart review study using anonymized data, study-specific informed consent was not obtained.

## Figures and Tables

**Figure 1:**
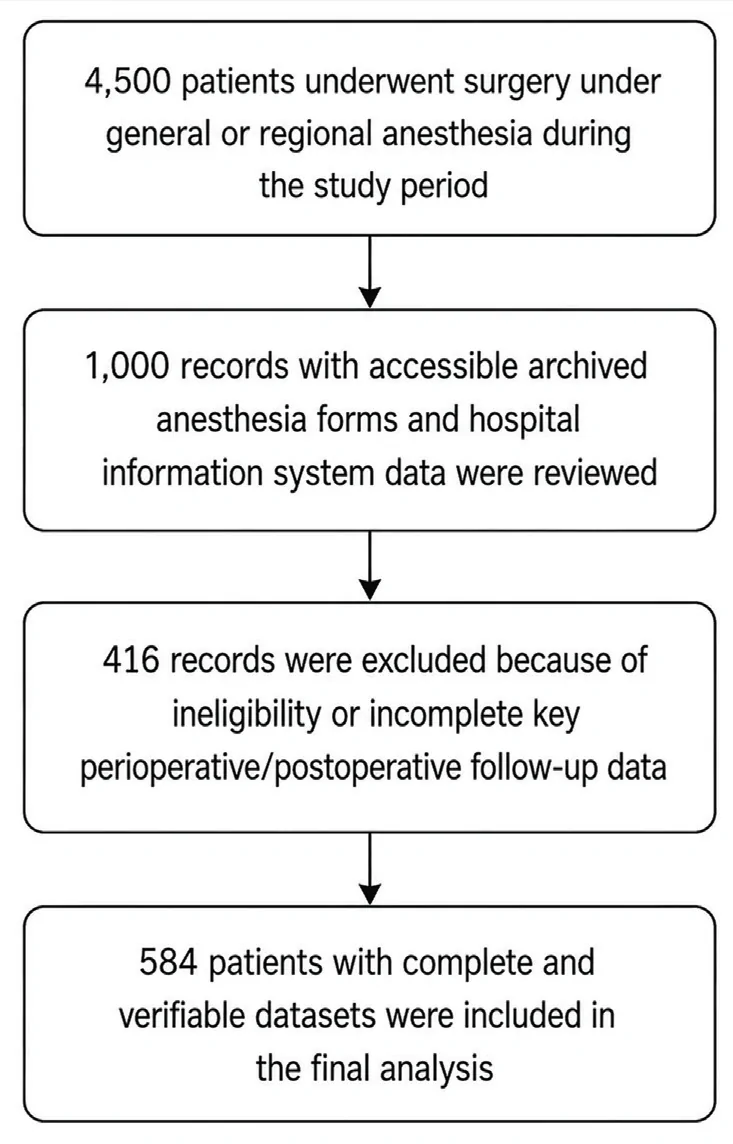
Patient flow diagram

**Table 1. Baseline Demographic and Perioperative Characteristics of the Study Population:** 

**Parameter**	** Mean ± SD**	
Age (years)	49.40±17.812	
Height (cm)	168.56±10.658	
Weight (kg)	76.55±14.692	
Duration of surgery (min)	117.05±70.863	
Duration of anaesthesia (min)	133.36±74.250	
Total blood loss (mL)	168.37±315.58	
Total fluid given (mL)	1757.55±1198.72	
**ASA**	**Frequency** **(n)**	**Percentage** **(%)**
I	114	19.5
II	403	69
III	67	11.4
Type of anaesthesia	-	-
General anaesthesia	461	78.9
Regional anaesthesia	123	21.1
Surgical risk level	-	-
Low-risk	286	49.0
Medium-risk	263	45.0
High-risk	35	6.0
Total	584	100.0

Values are presented as mean ± SD or n (%). n, number of patients; ASA, American Society of Anesthesiologists; SD, standard deviation

**Table 2. Surgical Characteristics of the Study Population:** 

**Type of Surgery**	**Frequency (n)**	**Percentage (%)**
Tonsillectomy	1	0.2
Rhinoplasty	23	3.9
Septoplasty	13	2.2
Tympanoplasty	5	0.9
Endoscopic sinus surgery	5	0.9
Ophthalmologic surgeries	8	1.4
Other head and neck surgeries	45	7.7
Abdominal-laparoscopic-lower	71	12.2
Abdominal-laparoscopic-upper	28	4.8
Abdominal-non-laparoscopic-lower	165	28.3
Abdominal-non-laparoscopic-upper	53	9.1
Thoracic-laparoscopic	6	1.0
Thoracic-non-laparoscopic	13	2.2
Extremity surgeries	118	20.2
Other surgeries	30	5.1
Total	584	100.0

Values are presented as n (%). n, number of patients

**Table 3. Baseline PONV Risk Factors and Anaesthesia-Related Exposures:** 

**Anesthetic agent (yes/no)**	**Frequency (n)**	**Percentage (%)**
Inhalation anaesthesia	428/156	(73.3/26.7)
Nitrous oxide	1/583	(0.2/99.8)
TIVA	26/558	(4.5/95.5)
Female/male	295/289	(50.5/49.5)
History of motion sickness (yes/no)	12/572	(2.1/97.9)
Non-smoker (yes/no)	333/251	(57.0/43.0)
Perioperative opioid use (yes/no)	331/253	(56.7/43.3)
**Apfel** **risk** **score**		
0	62	10.6
1	164	28.1
2	258	44.2
3	98	16.8
4	2	0.3
**Fasting** **duration** **(hours)**		
8	87	14.9
10	333	57.0
11	5	0.8
12	102	17.5
13	15	2.6
14	33	5.7
15	3	0.5
16	6	1.0
Total	584	100.0

Values are presented as n (%). n, number of patients; TIVA, total intravenous anaesthesia; PONV, postoperative nausea and vomiting

**Table 4. Mutually Exclusive Antiemetic Prophylaxis Regimens Used in the Study Population:** 

**Regimen**	**Frequency (n)**	**Percentage (%)**
No antiemetic prophylaxis	117	20.0
Ondansetron 4 mg only	147	25.2
Metoclopramide 20 mg only	163	27.9
Ondansetron+dexamethasone	134	22.9
Rare or overlapping prophylaxis regimens	23	3.9
Total	584	100.0

Values are presented as n (%). Rare or overlapping prophylaxis regimens included ondansetron+metoclopramide, ondansetron+metoclopramide+dexamethasone, ondansetron+scopolamine+dexamethasone, dexamethasone only, and metoclopramide+dexamethasone. n, number of patients

**Table 5. Incidence of Postoperative Nausea and Vomiting and Rescue Antiemetic use by Postoperative Time Point:** 

-	**PONV (yes/no)**	**Ondansetron/metoclopramide**
Postop 1^st ^hour	128/456	48/77
Postop 6^th^ hour	47/537	14/28
Postop 12^th^ hour	14/570	7/4
Postop 24^th^ hour	7/577	3/2

Values are presented as n. Rescue antiemetic values indicate recorded ondansetron and metoclopramide administration at each time point and may not equal the total number of patients with documented PONV. PONV, postoperative nausea and vomiting; Postop, postoperative

**Table 6. PONV-Associated Factors by Postoperative Time Point and Characteristics of Patients with Early PONV:** 

**Panel A. PONV-associated factors by postoperative time point**					
Variable	1^st^ hour	6^th^ hour		12^th^ hour	24^th^ hour
Type of surgery	0.157	0.020		0.889	0.034
Type of anaesthesia	0.001	0.146		0.174	0.169
ASA classification	0.466	0.813		0.888	0.773
Surgical size	0.501	0.004		0.586	0.346
Blood product use	0.408	0.041		0.624	0.917
Female gender	0.023	0.012		0.034	0.266
Motion sickness history	0.794	0.001		<0.001	0.700
Non-smoker	0.015	0.712		0.270	0.123
Perioperative opioid use	0.011	<0.001		0.560	0.751
Risk score	0.430	0.188		<0.001	0.062
Inhalation anaesthesia	0.021	0.222		0.441	0.108
TIVA	0.037	0.032		0.414	0.566
Fasting duration	0.367	0.205		0.730	0.998
High FiO_2_	0.374	0.780		0.696	0.915
NSAID use	<0.001	<0.001		0.624	0.032
Ondansetron use	0.001	0.019		0.681	0.294
Metoclopramide use	0.981	0.440		0.832	0.135
Dexamethasone use	0.024	0.907		0.121	0.516
**Panel** **B.** **Characteristics** **of** **patients** **with** **PONV** **during** **the** **first** **postoperative** **hour**					
**Demographic** **data** **of** **patients** **with** **nausea** **and** **vomiting** **in** **1** **hour**			**Value** **(mean** **±** **SD)**		
Age (years)			44.43±18.31		
Height (cm)			168.33±8.57		
Weight (kg)			76.39±15.15		
Duration of surgery (min)			134.53±80.89		
Duration of anaesthesia (min)			151.80±82.67		
Total blood loss (mL)			173.63±286.55		
Total fluid administered (mL)			1944.53±1293.93		
Gender (F/M) (%)			76/52 (59.4/40.6)		
ASA I/II/III (n) (%)			28/90/10 (21.9/70.3/7.8)		
Type of anaesthesia (general/regional)			114 (89.1)/14(10.9)		
Surgery type (laparoscopic/non-laparoscopic)			18 (14.1)/110 (85.9)		
Surgical risk (low/medium/high)			59 (46.1)/63 (49.2)/6 (4.7)		
RBC Transfusion (yes/no) (%)			4 (3.1)/122 (96.8)		
Motion sickness history (yes/no) (%)			3 (2.3)/125 (97.7)		
Non-Smoker (yes/no) (%)			85 (66.4)/43 (33.6)		
Perioperative opioid use (yes/no) (%)			58 (45.3)/70 (54.7)		
Ondansetron (%)			83 (64.8)		
Metoclopramide (%)			40 (31.3)		
Dexamethasone (%)			22 (17.2)		

Panel A values are presented as *P *values. Panel B values are presented as mean ± SD or n (%). n, number of patients; F/M, female/male; ASA, American Society of Anesthesiologists; TIVA, total intravenous anaesthesia; FiO_2_, fraction of inspired oxygen; NSAID, non-steroidal anti-inflammatory drug; PONV, postoperative nausea and vomiting; RBC, red blood cell; SD, standard deviation

**Table 7. Multivariable Logistic Regression Analysis for First-Hour PONV:** 

**Variable**	**Adjusted OR (95% CI)**	***P*** **value**
Female sex	1.28 (0.82-1.99)	0.272
Non-smoking status	1.38 (0.88-2.15)	0.158
Perioperative opioid use	0.58 (0.38-0.89)	0.013
General anaesthesia	1.62 (0.57-4.61)	0.366
Inhalational anaesthesia	1.36 (0.50-3.71)	0.551
Total intravenous anaesthesia	2.55 (0.83-7.85)	0.102
Metoclopramide-only prophylaxis	1.09 (0.41-2.89)	0.861
Ondansetron-only prophylaxis	2.05 (0.78-5.37)	0.146
Ondansetron+dexamethasone prophylaxis	0.84 (0.30-2.36)	0.743
Rare or overlapping prophylaxis regimens	1.77 (0.49-6.41)	0.385

Values are presented as adjusted odds ratios (ORs), 95% confidence intervals (CIs), and *P *values. The dependent variable was first-hour postoperative nausea and vomiting. No antiemetic prophylaxis was used as the reference category for antiemetic prophylaxis regimen variables. For binary variables, the reference categories were male sex, smoking status, no perioperative opioid use, regional anaesthesia, no inhalational anaesthesia, and no TIVA. PONV, postoperative nausea and vomiting; TIVA, total intravenous anaesthesia
